# Ramp Lesions in Chronic Anterior Cruciate Ligament Injuries

**DOI:** 10.7759/cureus.28450

**Published:** 2022-08-26

**Authors:** Asjad Mahmood, Sai Krishna MLV, Siva Srivastava Garika, Ravi Mittal, Vijay Kumar Digge, Shivanand Gamanagatti

**Affiliations:** 1 Orthopaedics, All India Institute of Medical Sciences, New Delhi, IND; 2 Radiology, All India Institute of Medical Sciences, New Delhi, IND

**Keywords:** anterior cruciate ligament, ramp lesions, posteromedial portal, epidemiology, trans-notch view, acl injury, meniscus ramp lesions

## Abstract

Background: Meniscus ramp lesions associated with anterior cruciate ligament (ACL) injuries are being increasingly reported in the literature. This study was carried out to know the incidence of ramp lesions in ACL injured patients and to study the characteristics of these patients in our population.

Methods: Seventy-five patients who underwent ACL reconstruction from January 2021 to December 2021 were prospectively studied. Patients with multi-ligament injuries or a history of previous knee surgery were excluded. All patients were examined clinically and all underwent MRI examinations. The findings of arthroscopy during ACL reconstruction were recorded and analyzed.

Result: Seventeen patients had ramp lesions with an incidence of 22.67%. Eight were isolated ramp lesions, and nine had other meniscus injuries. Ramp lesions were identified with 41.18% sensitivity using preoperative MRI. Thirteen out of 17 patients with ramp lesions had increased mobility of the posterior horn of the medial meniscus on anterior probing. The duration from injury to surgery was significantly longer in patients with ramp lesions as compared to patients without ramp lesions.

Conclusion: A ramp lesion is not an uncommon lesion in ACL injuries and can occur either as an isolated meniscus lesion or in association with other meniscus lesions.Ramp lesions can occur in road traffic accidents as well and are not just sports-related injuries. Ramp lesions are not visible through routine anterior portal diagnostic arthroscopy and their repair adds to the stability of the knee. The absence of ramp lesions on MRI does not rule out their presence; hence, one should always look for ramp lesions in the posteromedial compartment of the knee in all cases undergoing ACL reconstruction.

## Introduction

Anterior cruciate ligament (ACL) injuries are often associated with injuries to the other ligaments of the knee and menisci. Generally, acute ACL injuries present with lateral meniscus tears and chronic ACL injuries with medial meniscus tears [1]. The incidence of a medial meniscus tear in chronic ACL injury is 36-44% and that of a lateral meniscus tear is 22-35% [2,3].

The meniscus plays a pivotal role in maintaining biomechanical stability [4,5]. In ACL-deficient knees, the medial meniscus takes the part of a secondary stabilizer in preventing excessive anterior translation of the tibia and makes it more vulnerable to tear [6]. These tears were mainly seen at its posterior and peripheral attachments to the tibia near the posterior joint capsule.

An injury at the posterior meniscocapsular junction of the medial meniscus commonly associated with an ACL injury is called a ramp lesion. In 1982, Strobel first described it and used the term “ramp lesion” [7]. These meniscus tears are not visible during diagnostic arthroscopy by standard anterior portals because of their posterior location. Magnetic resonance imaging (MRI) scans can also fail to detect these tears more frequently, as compared to the tears that occur elsewhere in the medial meniscus. However, these meniscus ramp lesions, if undetected during ACL reconstruction, can lead to residual knee instability, increasing the graft's stress. Residual instability can also lead to the wearing out of articular cartilage in the medial compartment [8].

The purpose of this study is to determine the incidence of ramp lesions in ACL injured knees and to study the characteristics of patients with ramp lesions associated with an ACL injury.

## Materials and methods

Study design

This was a prospective study on the patients who underwent ACL reconstruction from January 2021 to December 2021. All skeletally mature patients who underwent primary ACL reconstruction during this period were included in the study. Patients with concomitant other ligament knee injuries and those who were scheduled for revision surgeries were excluded from this study. Informed consent was taken from the patients who were scheduled for arthroscopic ACL reconstruction. Institute ethical committee clearance was obtained from the All India Institute of Medical Sciences, Institutional Ethical Committee (IEC/605/7/2022).

Preoperative evaluation

The preoperative evaluation emphasized the collection of information regarding the duration and mode of injury to the knee. The knee examination included the range of motion, the Lachman test, the anterior drawer test, the pivot shift test, the posterior drawer test, the varus, and the valgus stress tests. All of these patients had anteroposterior and lateral radiographs of the knee along with an MRI. The knee MRI was discussed with a musculoskeletal radiologist to confirm the clinical findings.

Surgical technique

**Figure 1:**
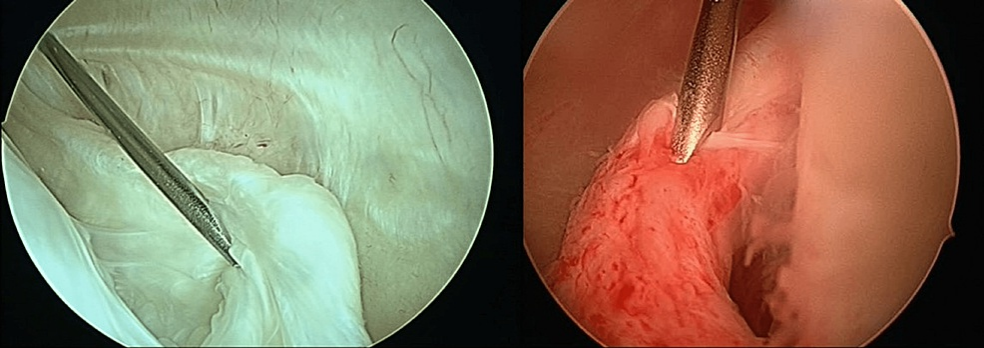
An 18G needle passed through the posteromedial portal site to identify the ramp lesion.

**Figure 2:**
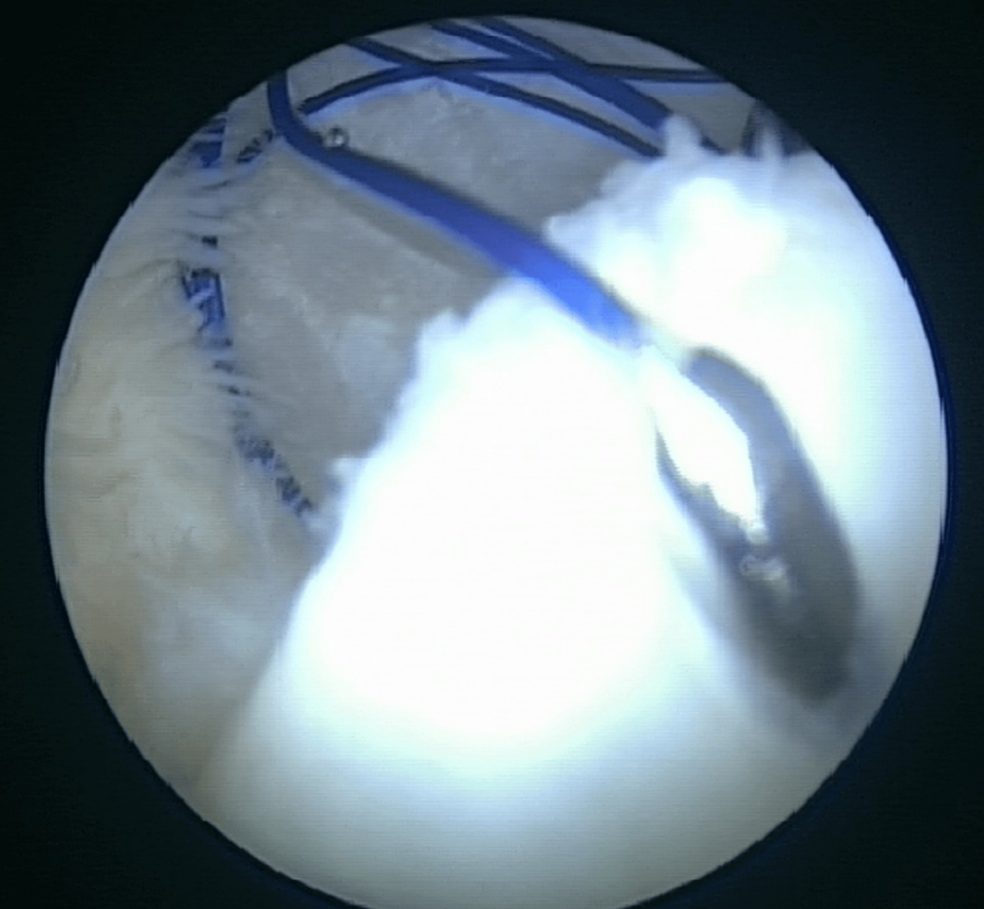
The Spectrum curved hook used to pass the Prolene suture in a vertical fashion across the ramp lesion.

**Figure 3:**
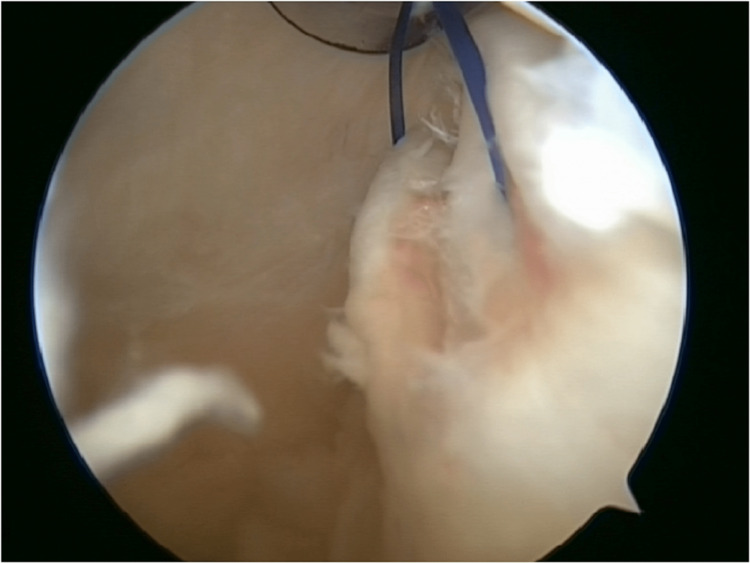
Both ends of the Prolene suture were taken out of the cannula in the posteromedial portal. It can be exchanged with Ethibond suture in a railroad fashion.

**Figure 4:**
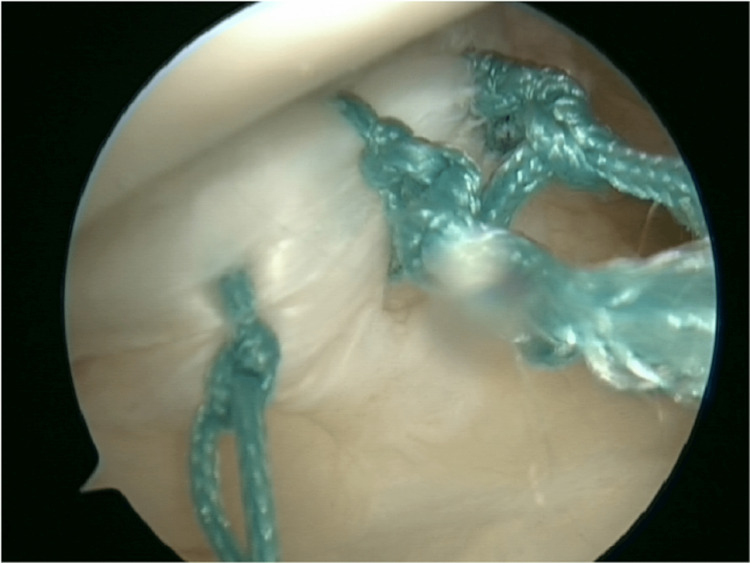
Complete repair of ramp lesion with three sutures.

Arthroscopic anatomic single-bundle ACL reconstruction was performed in all the patients using either a hamstring tendon (quadrupled) or a bone-patellar tendon-bone (BPTB) graft. Arthroscopy was performed using a 30° arthroscope. The anterolateral portal was used as a viewing portal, and the anteromedial portal was used for instrumentation. Besides confirming the ACL injury, the posterior cruciate ligament (PCL), articular cartilage, and both menisci were examined during arthroscopy. The mobility of the medial meniscus was checked with the help of a probe. Visualization of the posteromedial compartment was done using the trans-notch maneuver. This involves the insertion of the Wissinger rod in between the PCL and the lateral wall of the medial femoral condyle into the posteromedial compartment and advancing the scope over the rod. During this step, the knee was stabilized at 90° flexion. Internal rotation of the knee aided in the visualization of the posteromedial meniscus. An 18G needle was inserted through the soft spot located between the medial femoral condyle and proximal tibia at the posteromedial aspect of the joint line under arthroscopic visualization. It was used to probe the posterior meniscocapsular junction area to identify the ramp lesion (Figure 1). If the ramp lesion was not found at this stage, we proceeded with the reconstruction of the ACL and management of other meniscus lesions. A posteromedial portal was created if a ramp lesion was identified. An 8-mm cannula was placed in the posteromedial portal and the ramp lesion was visualized through this portal also. The tear was gently debrided using a shaver before the repair. We used a Spectrum® (Conmed, Utica, NY) hook loaded with a No. 1 Prolene® (Ethicon, Somerville, NJ) suture, which was passed through the cannula in the posteromedial portal for the repair of the ramp lesion. Using the Spectrum® device, the Prolene® suture was passed across the ramp lesion in a vertical fashion (Figure 2). Then, with the help of a suture retriever, both ends of the Prolene® suture were retrieved through the cannula in the posteromedial portal (Figure 3). This was exchanged with Ethibond® No. 2 suture (Johnson & Johnson Inc., New Brunswick, NJ) by the railroading technique. Both the ends of the Ethibond® suture were tied using a sliding knot followed by multiple alternating half hitches. Suture ends were cut using a suture cutter passed through the posteromedial cannula. The complete repair of the ramp lesion required one to three vertical sutures (Figure 4). The final repair was checked with a probe and was followed by the management of other meniscus lesions and ACL reconstruction.

Postoperative protocol

All the patients were given standard rehabilitation for ACL reconstruction, which employed isometric quadriceps exercises, closed chain knee exercises, half squats, balance board exercises, and gait training. Rehabilitation was not delayed when meniscus tears were repaired, except in situations where radial tears of either meniscus were repaired. All patients were followed up at two weeks, six weeks, and 12 weeks postoperatively.

Statistical analysis

All the data generated were tabulated in an Excel sheet (Microsoft Corporation, Redmond, WA) and analyzed using Stata software (Stata Statistical Software: Release 16; StataCorp LLC, College Station, TX). An independent t-test for continuous variables and a chi-squared (χ2) test for categorical data were used. P-values of less than 0.05 were considered statistically significant.

## Results

A total of 78 patients underwent primary ACL reconstruction during the study period. Three patients were excluded from the study as two of them underwent lateral collateral ligament (LCL) reconstruction along with ACL reconstruction and one underwent medial collateral ligament (MCL) reconstruction along with ACL reconstruction. So, a total of 75 patients were included in the study. The demographic details of the patients are presented in Table 1. The mean age of the patients was 28.32 years (range: 17-50 years). A total of 69 patients (92%) were males whereas six patients (8%) were females. Of the patients, 35 (46.675) were having injuries in their right knee while 40 patients (53.33%) were having injuries in the left knee. The commonest mode of injury was sporting activities in 35 patients (46.67%), followed by road traffic accidents in 27 patients (36%), and falls by other mechanisms in 13 patients (17.33%). The duration from injury to surgery was variable, with only four patients (5.33%) presenting acutely after the injury, i.e., within six weeks of the injury. A total of 15 patients (20%) presented between six weeks and six months post injury, and 56 patients (74.67%) presented with chronic instability, i.e., six months after the injury. Of the 56 patients with chronic instability, 19 patients presented after 24 months of injury (Table 1). The mean duration between injury and surgery was 18.31 ± 16.84 months and the median duration from injury to surgery was 12 months (interquartile range = 24.5 - 6 = 18.5) (range: four weeks to 74 months).

**Table 1:** Demographic details of the patients.

S. No.	Parameter	Number of patients	Percentage of patients
1	Gender		
	Male	69	92%
	Female	6	8%
2	Side of injury		
	Right	35	46.67%
	Left	40	53.33%
3	Mode of injury		
	Sporting injury	35	46.67%
	Road traffic accident (RTA)	27	36%
	Fall (other than sporting injury and RTA)	13	17.33%
4	Duration from injury to surgery		
	<6 weeks	4	5.33%
	6 weeks to 6 months	15	20%
	>6 months	37	49.3%
	>2 years	19	25.3%

Out of 75 patients, arthroscopy revealed meniscus tears in 52 patients (Table 2). Out of these 52 patients with meniscus tears, medial meniscus tears (including ramp lesions) were found in 42 patients (56%), while lateral meniscal lesions were present in 24 patients (32%). Seventeen patients had ramp lesions, which amounted to a 22.67% incidence in this study. Of these 17 cases with ramp lesions, eight had isolated ramp lesions without any other meniscus injury. In five patients, ramp lesions were associated with other lesions in the medial meniscus, i.e., medial meniscus tears other than ramp lesions (MMTOTRL). In two of the patients with ramp lesions, a radial tear in the body of the lateral meniscus was found. Two patients with ramp lesions had tears in the lateral meniscus and MMTOTRL. Cartilage degeneration in the medial compartment of the knee was found in eight patients (10.67%). The mean duration of injury to surgery in patients having medial compartment cartilage degenerative changes was found to be 36.37 months.

**Table 2:** Meniscus lesions as identified on arthroscopy. MMTOTRL: medial meniscus tear other than ramp lesion; MM: medial meniscus; LM: lateral meniscus.

Status of the meniscus as identified on arthroscopy		No. of patients	
No meniscus tear		23	
Medial meniscus tear	MMTOTRL	15	
	Isolated ramp lesion	8	
	MMTOTRL with ramp lesion	5	Vertical tear - 3; bucket handle tear - 1; vertical + horizontal tear - 1
Both MMTOTRL and lateral meniscus tear	Without ramp lesion	10	
	With ramp lesion	2	Case 1 - MM - vertical and radial tear + LM horizontal tear. Case 2 - MM - vertical tear + LM - horizontal tear.
Lateral meniscus tear	Isolated lateral meniscal tear	10	
	Lateral meniscal tear with ramp lesion	2	Radial tear = 2
Total		75	

Except for one female, all of the patients with an ACL injury and ramp lesion were men. The mean age of the patients with ACL injury with ramp lesion was 26 years (range: 17-47 years). At the same time, the mean age of the patients with an ACL injury without having a ramp lesion was 29 years. On applying an unpaired t-test, it was found that there was no statistical difference (p = 0.22) in age between the patients having ACL injuries with and without concomitant meniscus ramp lesion. Eight patients had an injury to their right knee, while nine patients had injured their left knee. Eight patients suffered an injury while playing or doing sporting activities, six patients suffered an injury in a road traffic accident, and three patients suffered an injury to the knee by sustaining a fall while walking or falling of heavy object on the leg. On applying the chi-square test, it was found that there was no statistically significant difference (p = 0.99) between the patients with and without ramp lesions for the mode of sustaining injury. The duration of injury to surgery in all the patients with ramp lesions was more than six months, with the mean duration of injury to surgery of 27.94 ± 12.66 months. The median duration of injury to surgery among patients with ramp lesions was 26 months (interquartile range = 36 - 20 = 16) (range: 8-48 months). In comparison, the mean duration of injury to surgery in patients without having a meniscus ramp lesion was 15.67 months. This difference in the time of the injury to the surgery between the ramp and without ramp lesion in ACL injured patients was found to be statistically significant (p = 0.0072).

On probing from the anterior portal, 13 out of these 17 patients with ramp lesions showed gross excessive mobility of the posterior horn of the medial meniscus even though the tear was not visible, while four of the patients had minimal excessive mobility of the medial meniscus. These could have been missed if the posteromedial compartment was not examined. All these patients with ramp lesions had complete separation of torn margins and were repaired with nonabsorbable sutures. During the repair of these lesions, 13 patients needed two vertical sutures, two patients needed three vertical sutures whereas, and in the remaining two patients, only one vertical suture was required. Cartilage degeneration over the medial femoral condyle in the form of grade 1 or 2 changes according to the Outerbridge classification was present in three of these 17 patients. Patients with ramp lesions having cartilage degenerative changes over the medial compartment of the knee were found to have an even longer duration of injury to surgery, with a mean duration of 40 months compared to 25.36 months in patients with ramp lesions without having cartilage degeneration. Only seven lesions were identified on the MRI, with a sensitivity of 41.18%. However, it was 100% specific with no false positives. No neurovascular or wound complications were encountered in any patient.

## Discussion

Meniscus ramp lesions are now being reported consistently in the literature, owing to increased awareness of their presence and significance. However, ramp lesions can easily be missed during arthroscopy from the anterior portals. This is due to inadequate visualization of the area around the posterior meniscocapsular junction. Probing for excessive mobility of the posterior horn of the medial meniscus is one method of detecting ramp lesions during arthroscopy via anterior portals. But this might not be the scenario in every case. Even if the posterior horn of the medial meniscus does not exhibit excessive mobility, it is possible to discover a ramp lesion when observing the posteromedial compartment. Trans-notch visualization of the posteromedial compartment is performed. But at times, the area around the posterior meniscocapsular junction is obscured by the capsular folds. Putting a needle through the posterior medial joint safe spot and probing with the needle tip helps alleviate this problem. Some authors routinely recommend a posteromedial portal for diagnostic arthroscopy [9,10]. In our view, adequate visualization of the ramp area is possible from the trans-notch view with a needle passed through a posteromedial soft spot acting as a probe. The posteromedial portal can be reserved for the repair of the ramp lesion when it has already been identified. The ramp lesion may occur as an isolated meniscus lesion or in association with other lesions in either meniscus. The presence of lateral meniscus lesions or MMTOTRL should not prevent the surgeon from actively looking for ramp lesions.

The incidence of ramp lesions in patients undergoing arthroscopic ACL reconstruction in this study was 22.67%. This figure is within the range reported previously in the literature, with incidence ranging from 9.3% to 42% [11-14]. The literature shows that the incidence of ramp lesions increases with the chronicity of the injury [13,15]. In this study, all 17 patients having ramp lesions had a chronic ACL injury, i.e., more than six months duration. A statistically significant difference was found in our study when comparing ACL injured patients with or without ramp lesions as far as injury duration was concerned.

There are no standard guidelines for the management of meniscal ramp lesions yet. The literature review shows contrasting views of the authors about the management of these meniscal tears. According to some authors, the lesion being in the peripheral red-red zone has good healing potential and the biology associated with ACL reconstruction also favors it and does not require repair [13]. On the other hand, some authors suggest that ramp lesions, regardless of whether they are stable or not, should be repaired. This is because, when unrepaired, mild anteroposterior laxity can adversely affect the biomechanical environment in the healing of a stable ramp lesion [16]. DePhillipo et al. found that the results of ramp repair along with ACL reconstruction were similar to those of isolated ACL reconstruction (where there was no ramp lesion) [17]. Recent research studies indicate that repair of the ramp lesion is indicated in chronic ACL instability [18-20]. In our study, all the ramp lesions were unstable and therefore repaired in all cases. Currently, there is no postoperative rehabilitation protocol described specifically for ramp lesion repair. General principles of rehabilitation after ACL reconstruction are followed even when it is accompanied by ramp lesion repair [21].

Low sensitivity of the MRI in detecting ramp lesions was observed, and hence its reliability in diagnosing ramp lesions was not high. In our study, preoperative MRI was only 41.18% sensitive in identifying ramp lesions. A recent study by Yasuma et al. [22] observed the sensitivity of MRI for detecting ramp lesions to be as low as 27.3%, while a study published in 2017 by Arner et al. [23] has shown the sensitivity of MRI for detecting ramp lesions to be as high as 84.6%. The reason for this low sensitivity is the supine position of the knee in full extension during MRI scans. This leads to a reduction of the meniscocapsular separation. Therefore, arthroscopic exploration is necessary to diagnose ramp lesions even when there is no suspicion based on MRI.

The strength of the study is that it has a very homogenous set of patients with no confounding factors. All the patients who underwent ACL reconstruction in one calendar year were included in this study. The limitation of this study is its small sample size. We conclude that the ramp lesion is not an uncommon lesion in ACL injuries. It can occur as an isolated meniscus lesion or in association with other meniscus lesions. An active effort should be made to look for ramp lesions in the posteromedial compartment of the knee in all cases undergoing ACL reconstruction.

## Conclusions

A ramp lesion is not an uncommon lesion in ACL injuries and can occur either as an isolated meniscus lesion or in association with other meniscus lesions. Ramp lesions can occur in road traffic accidents as well and are not just sports-related injuries. Ramp lesions are not visible through routine anterior portal diagnostic arthroscopy and their repair adds to the stability of the knee. The absence of ramp lesions on MRI does not rule out their presence; hence, one should always look for ramp lesions in the posteromedial compartment of the knee in all cases undergoing ACL reconstruction.
